# Rapid microbial diversification of dissolved organic matter in oceanic surface waters leads to carbon sequestration

**DOI:** 10.1038/s41598-020-69930-y

**Published:** 2020-08-03

**Authors:** Philipp F. Hach, Hannah K. Marchant, Andreas Krupke, Thomas Riedel, Dimitri V. Meier, Gaute Lavik, Moritz Holtappels, Thorsten Dittmar, Marcel M. M. Kuypers

**Affiliations:** 1 Max Planck Institute for Marine Microbiology, Celsiusstraße 1, 28359 Bremen, Germany; 2 IWW Water Centre, Moritzstr. 26, 45476 Mülheim an der Ruhr, Germany; 3 Present Address: Division of Microbial Ecology, Centre for Microbiology and Environmental Systems Science, University of Vienna, Althanstrasse 14 UZA I, 1090 Vienna, Austria; 4 Research Group for Marine Geochemistry (ICBM-MPI Bridging Group), at the Institute for Chemistry and Biology of the Marine Environment (ICBM), University of Oldenburg, Carl-von-Ossietzky-Str. 9-11, 26129 Oldenburg, Germany; 5 Helmholtz Institute for Functional Marine Biodiversity (HIFMB), University of Oldenburg, Carl-von-Ossietzky-Str. 9-11, 26129 Oldenburg, Germany; 6 Present Address: Alfred Wegener Institute Helmholtz Center for Polar and Marine Research, Am Handelshafen 12, 27570 Bremerhaven, Germany

**Keywords:** Biogeochemistry, Environmental sciences, Marine chemistry

## Abstract

The pool of dissolved organic matter (DOM) in the deep ocean represents one of the largest carbon sinks on the planet. In recent years, studies have shown that most of this pool is recalcitrant, because individual compounds are present at low concentrations and because certain compounds seem resistant to microbial degradation. The formation of the diverse and recalcitrant deep ocean DOM pool has been attributed to repeated and successive processing of DOM by microorganisms over time scales of weeks to years. Little is known however, about the transformation and cycling that labile DOM undergoes in the first hours upon its release from phytoplankton. Here we provide direct experimental evidence showing that within hours of labile DOM release, its breakdown and recombination with ambient DOM leads to the formation of a diverse array of new molecules in oligotrophic North Atlantic surface waters. Furthermore, our results reveal a preferential breakdown of N and P containing molecules versus those containing only carbon. Hence, we show the preferential breakdown and molecular diversification are the crucial first steps in the eventual formation of carbon rich DOM that is resistant to microbial remineralization.

## Introduction

Dissolved organic matter (DOM) in the oceans represents one of the largest carbon pools on our planet^1^. Part of this pool consists of so called labile DOM, which is characterized by a short half-life of hours to days. While labile DOM comprises less than 1% of the total oceanic DOM inventory, its cycling represents one of the largest annual turnovers of carbon on the planet (~ 25 gigaton C year^−1^)^2^. During its turnover, most labile DOM is remineralized by microorganisms to CO_2_, but a small proportion is transformed into more recalcitrant DOM which is eventually exported to the deep ocean where it is stored for millennia^3,4^. In recent years, research has focused on the mechanisms that lead to recalcitrance of the DOM pool^5–9^. These studies have revealed that DOM becomes difficult for microorganisms to metabolize because the individual compounds that comprise DOM either cannot be broken down, or are present at such low concentrations that the metabolic costs associated within their utilization prevents consumption^5,8,9^.

The transformation of labile DOM and the subsequent export of recalcitrant DOM to the deep ocean is particularly efficient in the oceanic gyres^10^, which are the largest biome on Earth^11^. In the oligotrophic gyres CO_2_ is mainly fixed into biomass by picoeukaryotic primary producers, and unicellular cyanobacteria such as *Prochlorococcus* and *Synechococcus*^12^. Subsequently labile DOM is released into the surrounding waters though sloppy feeding and grazing by zooplankton (mainly microzooplankton in the case of *Synechococcus* and *Prochlorococcus*), excretion from primary producers and viral lysis^13^. Upon its release, heterotrophic microorganisms transform and breakdown labile DOM, releasing nitrogen and phosphorus which are recycled in the oceanic gyres and sustain up to 85% of primary production in these oligotrophic regions^14^.

Despite the importance of DOM as a source of energy and nutrients in the oligotrophic gyres, little is known about the transformation and cycling that phytoplankton derived labile DOM undergoes immediately upon its release. Especially unclear is the role that rapid processing of labile DOM by marine prokaryotes plays in forming recalcitrant DOM. This is largely because the transformation and cycling of labile DOM is difficult to study due to it short residence time, low micro- to nano-molar concentrations, which are dwarfed by the high micromolar concentration of recalcitrant DOM, and the high molecular diversity of the DOM pool^13,15^. Stable isotope tracer studies, especially in combination with single cell or molecular techniques, have provided insights into bulk remineralization rates of DOM and allow turnover to be linked to specific microbial community members. For example, ^13^C and ^15^N isotope labelling experiments combined with nanoSIMS revealed that viral infection cause algae to leak organic matter prior to viral lysis^16^, while DNA stable isotope probing (SIP) can track the incorporation of ^13^C-labelled DOC substrates into bacterioplankton communities^17^. Such approaches however, provide little insight into changes in the molecular composition of the labile DOM pool.

For many years, molecular level determination of DOM was restricted to carbohydrates, amino acids, lipids and aminosugars. Many pioneering studies investigating how heterotrophs shape DOM composition used natural communities of marine bacteria and added defined DOM sources such as glucose^18,19^, or investigated the transformations of specific compounds within bulk DOM upon the addition of cultured microorganisms^20–22^. These studies revealed that heterotrophs rapidly consume DOM upon its release, forming new DOM compounds of a different molecular weight. Many observations also indicated that major changes already occurred in the DOM pool within 24 h, leading to the suggestion that more insights would be gained by increased sampling frequency^21^.

In recent years considerable steps toward understanding DOM at the molecular level have been gained from new high resolution analytical techniques such as Fourier Transform Ion Cyclotron Resonance Mass Spectrometry (FT-ICR-MS)^23,24^. FT-ICR-MS reveals the changes in the molecular and compositional diversity of DOM that occur due to biotic and abiotic reactions, so far however many studies have used long incubation times of weeks to months, which miss the rapid changes that labile DOM undergoes immediately upon its release^9,25^. In one of the few FT-ICR-MS studies carried out at time scales of less than a day, it was shown that DON and dissolved organic sulfur (DOS) are preferentially degraded over DOC in coastal waters^26^. Even with such approaches though, turnover of labile DOM may be masked as utilization or removal of molecules is likely balanced by the input of new molecules^27^, a problem which can be overcome by combining FT-ICR-MS with ^13^C labeling approaches.

In this study, we hypothesized that the cycling, breakdown and preferential uptake of N and P containing DOM molecules begins immediately upon its release from phytoplankton in the oligotrophic ocean, and might be responsible for the non-redfield composition previously determined for labile DOM in surface waters. To test this, we investigated the microbial breakdown and transformation of labile phytoplankton derived DOM in the oligotrophic ocean over short time scales (1 day) by combining in situ incubation experiments with ^13^C-labeled DOM with isotope ratio mass spectrometry and FT-ICR-MS. Additionally changes in the microbial community composition resulting from the addition of labile DOM were monitored. This allowed us to detect and investigate the rapid cycling of phytoplankton derived labile DOM by a natural assemblage of microrganisms against the high natural background of seawater DOM in the oligotrophic North Atlantic, a major area of DOM export.

## Results and discussion

### Instantaneous microbial remineralization of labile DOM

Oligotrophic surface water collected from the Cape Verde region was incubated with ^13^C-DOM from lyophilized *Spirulina* cells. This substrate was comprised of a complex mixture of cyanobacteria derived compounds, which are abundantly present in oligotrophic waters. Around 2 µM was added to the water, which is equivalent to the natural concentration of DOM that is expected to be released on a daily basis. To ensure that the addition of the substrate had the least impact on the ambient DOM composition, we induced a partial lysis of the cyanobacterial community (and subsequently the release of naturally derived DOM), by raising the incubation temperature after 4 h by 2 °C for 2 h.

No significant change in overall microbial cell numbers was observed during the course of the 28 h incubation (Fig. S1). Moreover, throughout the incubation the microbial community composition in the ^13^C-DOM addition experiment and the parallel experiments amended with ^13^C-DIC was similar (Fig. S2). The dominant bacterioplankton groups were the photosynthetic cyanobacterium *Prochlorococcus* spp., and the heterotrophic *Bacteriodetes*, *SAR11*, *Rhodobacteraceae*, *SAR86* and *SAR116* (Fig. S2), which is typical for microbial assemblages in oligotrophic surface waters^12, 17^. There was a transient increase of *Alteromonadaceae* until 16 h, after which *Rhodobacteraceae* abundance increased. The increase in relative abundance of these groups indicates that the induced partial lysis of the cyanobacteria community was successful as these groups of microorganisms are known to degrade and grow on cyanobacterial exudates and lysates^17^, and have previously been linked to the release of labile DOM associated with decaying phytoplankton blooms^16,26,28^.

**Figure 1:**
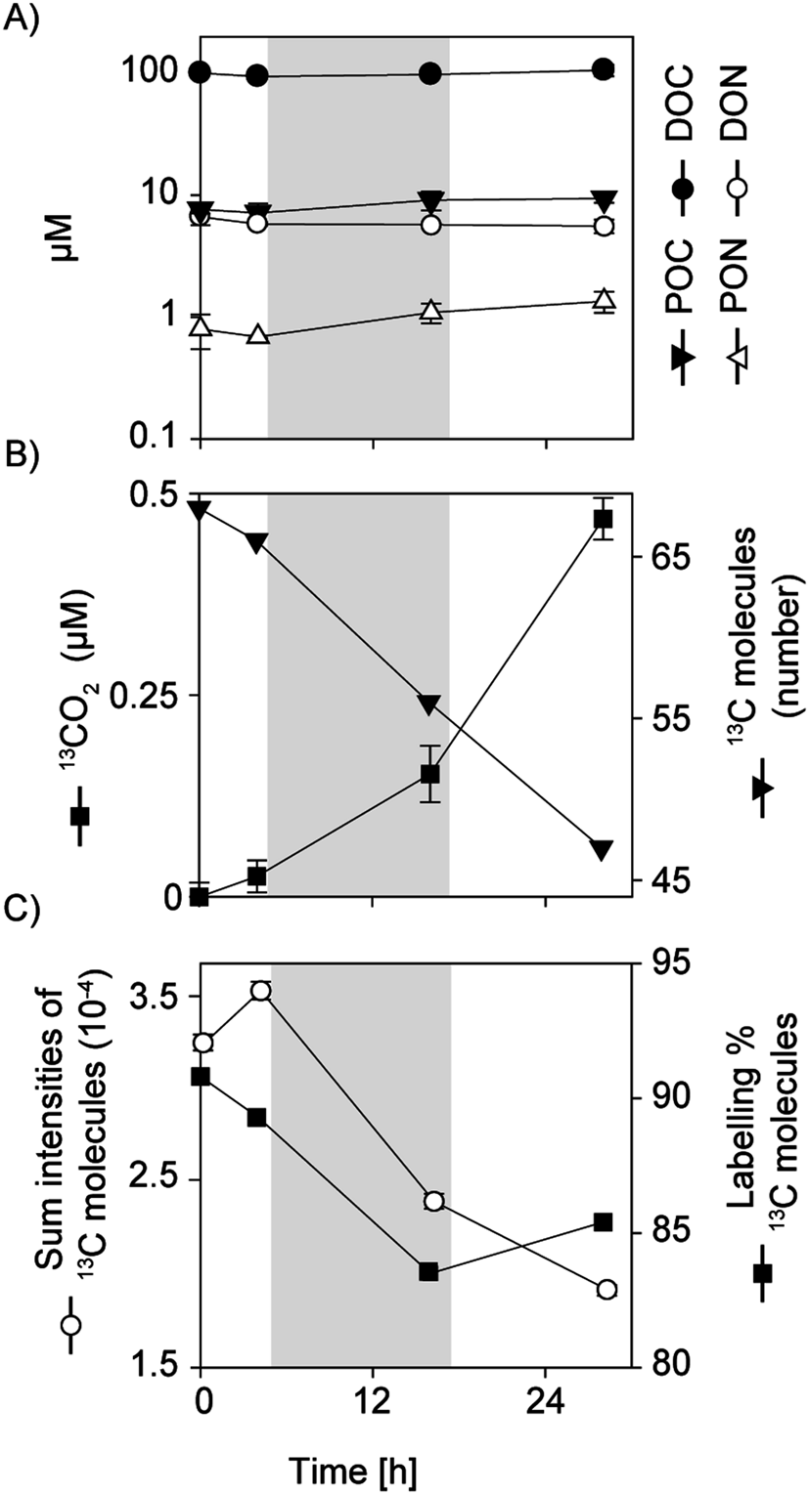
Changes in particulate and dissolved organic matter over time in oligotrophic surface waters (**A**) Organic matter concentrations in incubations amended with ^13^C-DOM (error bars are SD n = 3). (**B**) ^13^CO_2_ production from ^13^C-DOM (error bars are SD, n = 3) and number of DO^13^C molecules (**C**) summed normalized intensities of all DO^13^C molecules measured by the FT-ICR-MS and their average labeling percentage at each time point. The grey area denotes the night/dark period during the incubation. Note that the error bars are smaller than the bullets in many cases. POC = Particulate Organic Carbon, DOC = Dissolved Organic Carbon, PON = Particulate Organic Nitrogen, DON = Dissolved Organic Nitrogen.

The ^13^C-DOM addition was equivalent to 2% of the total DOM present and therefore mimicked the type and amount of relatively labile DOM released on a daily basis through viral lysis of cyanobacteria^12,29^. Throughout the incubation, average bulk particulate organic carbon and nitrogen concentrations remained the same (Fig. 1A), with a carbon to nitrogen (C:N) ratio of 8.5:1; higher than the Redfield ratio of 6.6:1, which is typical for phytoplankton biomass throughout the marine realm^30^. This offset is most likely a result of non Redfieldian uptake of nutrients by cyanobacteria, which has been observed previously in N-limited regions^31^. The C:N ratio of the DOM was around 17:1 (Fig. 1A), which is similar to the globally averaged stoichiometry of bulk DOM in the surface ocean^32^. This indicates that the bulk DOM was substantially depleted in nitrogen relative to the ratio of 10:1 previously suggested for labile DOM^4,33^. Similar observations have been made before in the Cape Verde region, as well as in the surface waters of the nitrogen limited east Japan Sea (14–20:1), and have been attributed to dissolved organic nitrogen utilization for both recycled primary and secondary production^34,35^.

While no significant changes in the bulk concentrations of particulate and dissolved organic C and N were observed during the incubation, a significant production of ^13^CO_2_ was already detected after 4 h, indicating rapid microbial remineralization of the ^13^C-DOM substrate (Fig. 1B). Remineralization to ^13^CO_2_ continued over the course of the incubation and corresponded to an average remineralization rate of ~ 0.5 µM ^13^C-DOM d^−1^, which was ~ 25% of the labile organically bound carbon we added. This does not take into account the utilization of ambient ^12^C-DOM, and therefore most likely represents a conservative estimate of the total remineralization rate. Nevertheless, the microbial remineralization rate was in the same order of magnitude as previously reported from phytoplankton blooms in the North Atlantic^36^. Moreover, the measured ^13^CO_2_-production rate was similar to the CO_2_ fixation rate of 0.6 µM d^−1^ (Fig. S1) and comparable to photosynthesis rates previously reported for this region^37^. Based on these rate measurements, nutrients released upon DOM remineralization supported more than 90% of the primary production in the oligotrophic surface waters, which agrees with current estimates that recycling of nutrient N and P from DOM mainly sustains algal growth in the oligotrophic oceans^14^.

### Rapid recombination of DOM leads to molecular diversification

Molecular level changes within the labile ^13^C-DOM pool were resolved using Fourier Transform Ion Cyclotron Resonance Mass Spectrometry for molecules within a mass range of 90–391 Da (Fig. S3–S5). The upper limit for mass detection was set to ensure unambiguous identification of ^13^C containing molecular formulae. These molecules are particularly relevant to the microbial community, as molecules up to a mass of ca. 600 Da can be taken up directly by microorganisms, rather than being degraded extracellularly^38^. Furthermore, only molecular formulae that had a ^13^C-labelling above 50% (i.e. more than 50% of the individual carbon atoms within the molecular formula were ^13^C) were included in subsequent analysis, this conservative threshold ensured that no molecules present in the ambient seawater were analyzed.

A total of 106 discrete ^13^C-DOM molecular formulae were identified (Table S1). The abundance of the observed compounds decreased throughout the incubation (Fig. 1B), concomitant with an increase in ^13^CO_2,_ indicating that the added ^13^C-DOM substrate was being remineralized already within the first 4 h by microbial activity. The summed normalized intensity of the detected molecular formulae rose slightly between the 0 h and 4 h and then decreased over time to almost half the 0 h value at 28 h (Fig. 1C). The initial increase in summed intensity indicates that “new molecules” from the originally added ^13^C-DOM entered the FT-ICR-MS mass window (90–391 Da). These likely stemmed from the degradation of ^13^C-DOM molecules larger than 391 Da within the first 4 h of the incubation.

**Figure 2:**
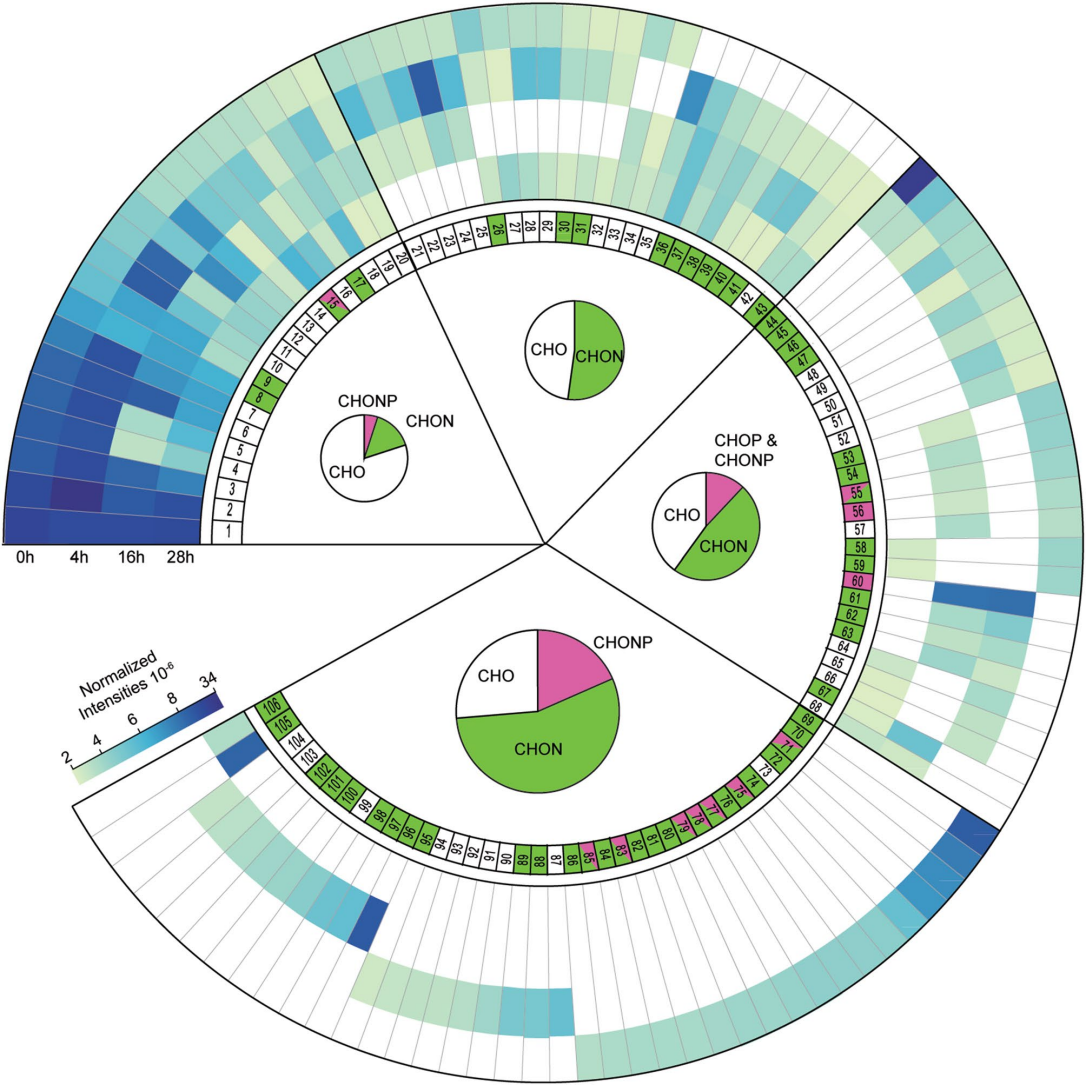
Rapid breakdown and transformation of labile ^13^C-DOM molecules in oligotrophic surface waters. Outer ring: Heatmap of the normalized FT-ICR-MS intensities of all detected ^13^C labelled molecules during the incubation. ^13^C labelled molecules are grouped based on their presence at all, three, two or one time point. Inner ring: Heteroatom composition of each molecular formula; colours represent molecular formulas only containing CHO (white), containing nitrogen (green), phosphorus (magenta) or nitrogen and phosphorus (green and magenta). Centre panel: Relative abundance of molecules based on heteroatom composition, where the size is representative of the total number of molecules present at all, three, two or one time point. See Sup. Table 1 for the molecular formulas of the 106 compounds. Note the axis break in the linear color scale of the normalized intensities.

The normalized intensity of most individual compounds did not show clear trends during the incubation (Fig. 2). Instead, some molecules increased, or increased and then decreased over time (Fig. 2). Over the course of the incubation, we continued to observe the appearance of new ^13^C-DOM molecular formulae in the 90–391 Da mass window (Fig. 2). These were also were most likely breakdown products of molecules with a mass higher than 391, rather than breakdown products of molecules that were already in the 90–391 Da mass window at 0 h as there was no trend towards the formation of smaller molecules in 90–391 Da mass window during the incubation (Fig. S6). The appearance of new molecules within 4 h indicates that this breakdown of larger labile DOM molecules occurs quickly in oligotrophic surface waters.

Remarkably, our results also indicate that the microbial community was recombining and altering the amended DO^13^C. There was a reduction in ^13^C-labelling percentage of the individual molecular formulae over the course of the incubation. As the molecules in question still have a labelling percentage far above natural abundance (> 50%) we are certain that the decrease in labelling percentage was due to alterations of our initially added substrate. The ~ 15% reduction in ^13^C-labelling within the DOM pool (Fig. 1C) cannot be attributed to carbon isotope fractionation during remineralization, as this would have a negligible effect on the ^13^C-content of the DOM molecules (^13^C-content changes in per mill rather than percent), and would rather enrich than deplete the DOM pool in ^13^C. In fact, as the majority of the molecules detected from the initially added substrate were ~ 100% labeled, the only way in which the labeling % of these molecules could have decreased was through recombination with compounds containing ^12^C from the ambient DOM. This recombination of organic molecules took place in a time span of only hours after the addition of ^13^C-DOM to the seawater. Currently, we can only speculate on the mechanisms behind the recombination of the labile substrate and the ambient DOM. Recent studies have shown that the exometabolomes of cultures organisms contain thousands of compounds which are not predicted by their metabolic pathways^7,39^. Many of these compounds are metabolites and biosynthetic precursors utilized by other (auxotrophic) microbes^40^. Thus, it is possible that recombination with ambient DOM occurred as enzymatic byproducts. Other processes such as cross linking via photo-oxidation could also play a role^41^.

Taken together, the decrease in the number of detectable ^13^C-labelled molecules, normalized intensities and labelling percentage during the incubation (Fig. 1B,C), show that ^13^C-DOM was rapidly degraded, recombined and also remineralized to ^13^CO_2_. Our combined results demonstrate that labile DOM in oligotrophic waters was broken down and transformed into new molecules in a complex manner over the course of a day. So far, the published data have indicated that this may be the case but were based on incubations lasting days, weeks or even years and have not shown that such molecular diversification happens on such rapid time scales^9,25^.

### Preferential breakdown of organic phosphorus and nitrogen

The H:C and O:C ratios of the ^13^C-labelled molecules did not change over the course of the incubation (Fig. S7). However, there were distinct changes in the heteroatom content of the molecules over time, indicating that N and P containing DOM molecules were preferentially broken down in the oligotrophic surface waters (Fig. 2). While most compounds containing only C, H and O were present at three or four time points, most P containing molecules were present only at the beginning of the incubation, and were broken down within 4 h (Fig. 2). N containing molecules were also rapidly broken down, with most N-containing molecules present at only one or two time points (Fig. 2). These results indicate that dissolved organic P and N are preferentially broken down over dissolved organic C in the oligotrophic surface waters.

The preferential breakdown of dissolved organic P and N has been suggested previously based on the elemental ratios of DOM observed for surface and deeper waters^34,42^, and based on observations from weeks to months long incubations^33,43^. In situ measurements showed that recalcitrant DOM has a higher of DOC:DON:DOP ratio (3,511:202:1) than labile DOM (199:20:1)^42^, and the long incubation studies indicated that this was a consequence of DOP being degraded preferentially followed by DON and then DOC^33^. Our results show that the microbial community instantaneously starts altering the C:N:P ratios of DOM by preferentially breaking down P and N containing organic molecules in oligotrophic waters. This has implications for our understanding of elemental ratios of labile DOM and its cycling. So far, marine labile DOM was believed to have an elemental ratio of C:N:P of 199:20:1^42^, divergent from the Redfield ratio ascribed to fresh phytoplankton biomass (106:16:1). Based on our observations we hypothesize that the elemental ratio of labile DOM does not differ from Redfield and that previously reported C:N:P ratios of labile DOM had already been influenced by microbial transformation. While this may seem intuitive, many earth system models which include regenerated production driven by DOM remineralization (e.g.^4,44,45^) do not take it into account and use a ratio around 199:20:1. Hence, they could be underestimating nutrient recycling in the surface waters by as much as 50% and therefore underestimate the amount of carbon exported to the deep ocean.

## Conclusions

Previously, analysis of DOM from different aquatic environments has shown that the diversity of DOM increases over time^8,46^. As a result, DOM becomes more recalcitrant, because individual compounds are present at vanishingly low concentrations and because certain compounds seem resistant to microbial degradation^5,8,47^. The increasing diversity and recalcitrance of DOM has been attributed to repeated and successive processing of DOM by microorganisms over time scales of years to millennia^4^. Until now however, the impact that microbial transformations have on DOM immediately upon its release into the water column have rarely been studied. Our data directly show that upon DOM release, a diverse array of new molecules is immediately formed by microbial breakdown, transformation and recombination in the oligotrophic surface waters. At the same time, it appears as that the new DOM molecules are already depleted in organic phosphorus and nitrogen within hours of DOM release. Together these transformations mark the first step that eventually leads to the formation of recalcitrant DOM. The export of this recalcitrant DOM has led to the accumulation of ~ 662 Gt of dissolved organic carbon in the deep ocean^1^. Consequently, the immediate diversification of DOM that already occurs in the surface waters leads to the sequestration of carbon in the deep ocean for millennia, partly counteracting anthropogenic carbon emissions.

## Materials and methods

### Field location and sampling procedure

Surface seawater (5–10 m) was collected during a cruise near the Cape Verdean Islands (16° 45′ N, 25° 08′ W) onboard the R/V Islandia on 30.9.2012. Approximately 120 L of seawater was collected shortly before dawn (5–6 AM) using 5 L niskin bottles attached to a CTD-rosette. Sampled water was transferred into forty 2.75 L polycarbonate Nalgene bottles without pre-filtration, which were previously acid-cleaned (HCl) and rinsed three times with ultrapure water. These incubation bottles were filled headspace-free and kept in the dark until further processing at the onshore laboratory within 4–6 h of sampling.

### Incubation experiments

In order to monitor the utilization of DOM in seawater, we added trace amounts (2 µM) of ^13^C-labeled DOC in form of lyophilized (freeze-dried) *Spirulina* cells (99% ^13^C-labelled, Campro Scientific). This substrate was the most representative substrate available that was (1) cyanobacterial-derived and (2) highly labeled in ^13^C. The use of a cyanobacterial derived substrate was required, as these are the dominant primary producers in the study region and it has been shown that heterotrophic bacteria can respond differently to DOM derived from cyanobacteria and diatoms^48^. Furthermore, previous studies have indicated that organic carbon added from freeze-dried *Spirulina* is degraded via the same pathways as ambient organic carbon in marine environments^49^. Shortly before the addition, lyophilized *Spirulina* cells were dissolved in milli-Q (0.081 g in 5 ml) and filtered through a 0.7 µM pre-combusted GF/F filter to remove any particulate matter. The DO^13^C concentration of the filtrate was 50 mM. Amendment incubation experiments were carried out by adding 100 µl of the DO^13^C substrate (2 µM final concentration), whereas controls were amended with 250 µM ^13^C dissolved inorganic carbon (DI^13^C) as described in Ref.^37^ (approximately 10% labeling percentage). Incubation bottles were placed in a continuous flow through water chamber, mimicking the natural temperature (26–28 °C) and sun irradiance levels of the surface ocean.

Subsets of incubation bottles were harvested at four different time-points over the course of 28 h in order to acquire a high resolution profile of the temporal utilization of DO^13^C. Time point zero (0 h) was sampled directly after the addition of the isotopes at midday, followed by samples before dusk (4 h), before dawn (16 h) and before dusk on the following day (28 h). After 4 h, the temperature of the water bath was raised to 30 °C for 2 h to induce a partial cell lysis of the cyanobacterial community, this mimics the partial lysis that occurs in situ during viral lysis events and was intended to release naturally derived DOM during the incubations, thus minimizing any effect of adding the ^13^C-DOM to the overall DOM composition and microbial community structure.

At each time point, five bottles were sacrificed for both the DO^13^C and DI^13^C amendment and samples were taken for the determination of the following parameters; cell counts, 16S rRNA, DI^13^C, NOx, particulate organic C (POC), ^13^C (PO^13^C) and N (PON) uptake, DOM for FT-ICR-MS, DOC and total nitrogen (TN), details for each analysis are given below.

### DNA extraction and 16S rRNA gene tag sequencing

Subsamples for DNA (1.25 L) were taken at each time point, filtered onto 27 mm Durapore filters (0.2 µm pore size; Millipore, Darmstadt, Germany) and stored at − 20 °C until further processing. DNA was extracted using the MoBio Ultra Clean Soil DNA extraction kit (MoBio Laboratories, Inc., CA, USA).

The V3 and V4 variable regions of the 16S rRNA gene were amplified using the primer combination Bakt_341F and Bakt_805R^50^. Barcoding of the samples was done by a six nucleotide sequence at the 5′-prime end of the forward primer. (Bakt_341Fmultiplex: 5′-barcode-CCTACGGGNGGCWGCAG-3′,Bakt_805R: 5′-GACTACHVGGGTATCTAATCC-3′). Five independent 50 µl PCR reactions were performed on each sample. Each of the five PCR reactions was composed of 1–3 µl of template DNA in H_2_O, 0.4 µM of each primer, 50 µM of each dNTP, and 1 U Phusion DNA polymerase (Thermo Fisher Scientific, Waltham, MA USA) in one fold concentrated Phusion DNA polymerase HF buffer with 750 µM MgCl_2_ and 5% (v/v) dimethyl-sulfoxide. The thermocycling was performed as follows: 30 s denaturation at 98 °C, followed by 28 cycles of 10 s denaturation at 98 °C, 30 s annealing at 55 °C and 15 s elongation at 72 °C and a 10 min final elongation step at 72 °C. The generated amplicons were size selected and purified by agarose gel electrophoresis and the QIAQuick Gel/PCR purification kit (QIAGEN, Venlo, Netherlands). Barcoded amplicons from different samples were pooled in equimolar concentrations. An Illumina True-Seq library was constructed from the amplicon pool and sequenced on an Illumina MisSeq sequencer in 2 × 300 bp mode at the Max Planck Genome Centre in Cologne, Germany.

Raw sequences were checked for adaptor remains with BBduk (https://sourceforge.net/projects/bbmap/). Resulting merged reads were split by barcodes using the Mothur software^51^. PCR and sequencing errors were removed and exact sequence variants were identified using DADA2 R-package^52^. Unique sequence variants were further clustered into larger OTUs using SWARM v. 2^51^. The SWARM representative sequences were aligned to SILVA SSU132 SEED database^53^ and classified by Last Common Ancestor approach by SILVA incremental aligner (SINA v1.2.11)^54^.

### Cell counts

Subsamples for 4′,6-Diamindino-2-Phenylindole (DAPI) cell counts (50 mL) were fixed in 1% formaldehyde for 1 h at room temperature, then filtered on a 0.2 µm polycarbonate filter and stored at − 20 °C. All cells were stained with DAPI for total cell abundances and were counted manually on an epifluorescence microscope (Axio Imager D.2, Zeiss). For each sample, at least 800 cells were counted for quantification of cell numbers.

### Gas–isotopic ratio mass spectrometry

DI^13^C concentrations were determined by collecting 12.5 mL subsamples headspace-free into Exetainers (Labco Ltd, High Wycombe, UK). 0.1 mL of a saturated HgCl_2_ solution was added to terminate biological activity, and samples were stored in the dark until analysis. DI^13^C production was measured by transferring a 1 ml subsample into an 12 ml exetainer. The headspace of 11 ml was flushed with helium, and then the sample was acidified with 100 µl of 85% orthophosphoric acid. The sample was analyzed using a Finnigan GasBench II attached to isotope ratio mass spectrometry (IRMS; Finnigan Delta Plus).

### POC and PON measurements

Bulk DI^13^C uptake rates and POC and PON concentrations were determined from triplicate incubation bottles from each time point as described in Ref.^37^. Briefly, 2.75 L of seawater were filtered onto pre-combusted (450 °C, 6 h) 25 mm GF/F filters (Whatman, St Louis, MO, USA) and stored at − 20 °C until further processing. Filters were acidified with fuming HCl in a desiccator overnight, oven dried for 1 h at 55 °C and folded in tin cups. Particulate organic C and N as well as the relative abundances of each isotope (i.e. ^12^C/^13^C) was determined by a Thermo Flash EA 1,112 elemental analyzer coupled to an isotopic ratio mass spectrometer (Finnigan Delta Plus XP, Thermo Fisher Scientific). CO_2_ fixation rates were subsequently calculated from the incorporation of ^13^C into biomass according to Ref.^37^.

### FT-ICR-MS method development

Over the course of the incubation, we analyzed changes in the molecular composition of the DO^13^C pool using Fourier Transform Ion Cyclotron Resonance Mass Spectrometry (FT-ICR-MS), focusing on masses between 90–391 DA (Fig. S3). To verify our ability to detect and measure molecules with very high ^13^C-labelling percentages on the FT-ICR-MS, measurements were carried out on a solid phase extracted (SPE) subsample of the pure DO^13^C Spirulina substrate, which was utilized in the experiments (Fig. S4). To check if ^13^C assigned peaks interfere with ^12^C peaks we mixed the SPE-DO^13^C from the *Spirulina* 1:1 with deep sea SPE-DOM (North Equatorial Pacific Intermediate Waters, NEqPIM). The ^12^C and ^13^C peaks could be clearly distinguished from each other in the mixture of SPE-DO^13^C and deep sea SPE-DOM.

Similarly, in the incubations, where the added DO^13^C comprised only 2% of the entire DOC pool, ^13^C-peaks could be clearly distinguished from the background DOC peaks (Fig. S4, S5). For example, at T0, a 100% ^13^C-labelled DOC peak with mass of 252.157 was visible in the amended sample but not the control sample. The same peak was not present anymore in the amended sample after 4 h of incubation. This demonstrates that this new method can detect the dynamic changes that occur within the DO^13^C fraction over short time periods, despite the high natural DOM background.

### Dissolved organic matter analysis

For quantification of dissolved organic carbon (DOC) and total nitrogen (TN), triplicate aliquots of 20 mL were taken at each time point. The aliquots were filtered through a pre-combusted (450 °C, 6 h) glass fiber filter (Whatman, St Louis, MO, USA). The pH of the filtrates were adjusted to pH 2 by adding HCl (32%, ultrapure) and frozen at − 20 °C for storage until further processing.

Dissolved organic carbon was analyzed as non-purgeable organic carbon by high temperature catalytic combustion using a Shimadzu TOC-VCPH/CPN instrument. Total dissolved nitrogen was detected using a TNM-1 module. Accuracy and precision were checked to be less than 5% using Deep Atlantic Seawater reference material (DSR, D.A. Hansell, University of Miami, Florida, USA). TN values were used to quantify DON in this study as NOx and NH_4_ were below the limit of detection in the oligotrophic surface waters.

The 0.5 L DOM samples taken at each time point were filtered, acidified and stored in the same manner as the DOC and TN aliquots.

The DOM was isolated from filtered and acidified subsamples by solid phase extraction (SPE) using commercially available modified styrene divinyl benzene polymer cartridges (PPL, Agilent, USA;^55^. After extraction, cartridges were rinsed with 0.01 M HCl to remove remaining salts, dried by a stream of Ar gas and eluted in 6 mL of methanol (HPLC-grade, Sigma-Aldrich, USA). The DOM extracts were stored in amber vials at − 20 °C until further analysis. The SPE method is the most efficient method available to isolate DOM from seawater and completely desalt it. Extraction efficiencies in this study was on average 53%, in comparison ultrafiltration recovers < 20% of DOC. A higher efficiency is gained using reversed osmosis/electrodialysis (RO/ED) (~ 80%), but does not remove salt and therefore is unsuitable for MS methods. Furthermore, SPE is widely used for metabolite analysis and can detect > 10,000 molecular formulae of small hydrophilic compounds during analysis of microbial exometabolites^7^. The extracts were measured in a 1:1 mixture of ultrapure water and methanol (HPLC-grade, SigmaAldrich, USA) on a 15 T Solarix FT-ICR-MS (Bruker Daltonics, USA) equipped with an electrospray ionization source (Bruker Apollo II) in negative ionization mode. Samples were analyzed in five mass spectral windows (Fig. S3) by applying a mass filter in the quadrupole unit of the Solarix system prior to detection in the ICR cell. The total mass windows covered the range m/z 90–515. A total of 250 scans were accumulated per run. Each sample was measured in triplicate.

The triplicate measurements of each sample were used to reduce the noise of the measurement^56^. The spectra were calibrated with an internal calibration list using the Bruker Daltonics Data Analysis software package. Molecular formulas were assigned to the mass peaks using a Matlab script modified from Ref.^57^ and considering the elements H, O, N, P and S and a mixture of ^12^C and ^13^C atoms (0–100% labeling of ^13^C). The script was run in succession; the first run calculated only ^12^C atoms and the second run included ^13^C atoms for the remaining mass peaks. Only peaks with a single formula assigned and a mass accuracy of < 0.3 ppm in at least 2 out of 3 runs were considered. This approach is highly conservative, especially in the higher mass range (> 350 Da) where the number of multiple assignments increased. Further investigation only involved molecules in which 50% or more of the carbon atoms were ^13^C atoms. This conservative approach gave us confidence that these molecules, due to their high deviation from natural abundance labeling percentage (1.1%), are derivatives from the added DO^13^C substrate (which was 99% ^13^C-labeled) and not natural products. In comparison, the standard analysis of natural organic matter samples usually only takes formulas with one additional ^13^C atom into account^23^. Mass spectra of each run were normalized to the sum of the peak intensities from all windows for that sample. Furthermore, at each time point, the DO^13^C amended samples were compared to the zero hour time point from the DI^13^C control, with the intention of removing any matching peaks from further analysis. However, none of the DI^13^C control peaks were more than 2% labeled. Therefore we are confident that the peaks used in the analysis were derived from the added ^13^C-DOM. The normalized intensities of the triplicate measurements were averaged for comparison between different samples.

## Supplementary information

Supplementary Information.

## Data Availability

All data is available in the main text or the supplementary materials.
