# Supplementary material for: Rapid microbial diversification of dissolved organic matter in oceanic surface waters leads to carbon sequestration
Source: Sci Rep. 2020 Aug 3;10:13025. doi: 10.1038/s41598-020-69930-y (PMC7400608; doi:10.1038/s41598-020-69930-y)
Supplement: Supplementary file 1 — Supplementary Information. [file 41598_2020_69930_MOESM1_ESM.docx]

**Supporting Information for**

Rapid microbial diversification of dissolved organic matter in oceanic surface waters leads to carbon sequestration

**Authors:** Philipp F. Hach^1^, Hannah K. Marchant^1^, Andreas Krupke^1^, Thomas Riedel^2^, Dimitri V. Meier^3^, Gaute Lavik^1^, Moritz Holtappels^1,#^, Thorsten Dittmar^4,5^ & Marcel M. M. Kuypers^1*^

**Affiliations:**

^1^Max Planck Institute for Marine Microbiology, Celsiusstraße 1, 28359 Bremen, Germany.

^2^IWW Water Centre, Moritzstr. 26, 45476 Mülheim an der Ruhr, Germany.

^3^Division of Microbial Ecology, Centre for Microbiology and Environmental Systems Science, University of Vienna, Althanstrasse 14 UZA I, 1090 Vienna, Austria.

^4^Research Group for Marine Geochemistry (ICBM-MPI Bridging Group), at the Institute for Chemistry and Biology of the Marine Environment (ICBM), University of Oldenburg, Carl-von-Ossietzky-Str. 9-11, 26129 Oldenburg, Germany.

^5^Helmholtz Institute for Functional Marine Biodiversity (HIFMB), University of Oldenburg, Carl-von-Ossietzky-Str. 9-11, 26129 Oldenburg, Germany.

^#^Present address: Alfred Wegener Institute Helmholtz Center for Polar and Marine Research, Am Handelshafen 12, 27570 Bremerhaven, Germany.

*Correspondence to: [mkuypers@mpi-bremen.de](mailto:mkuypers@mpi-bremen.de)


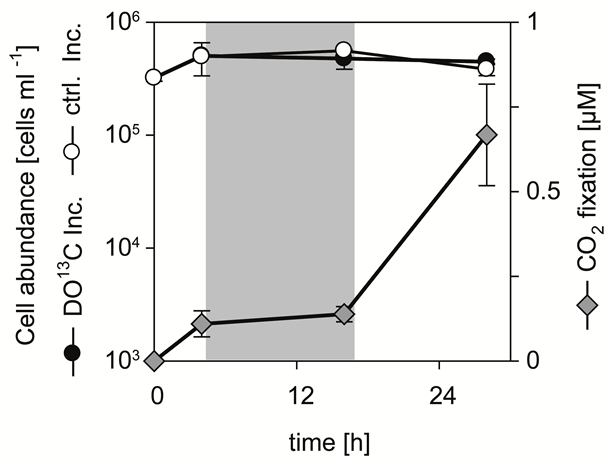


**Fig. S1.** Cell counts of the DO^13^C and DI^13^C control incubation and the CO_2_ fixation measured from the DI^13^C uptake in the control incubation. The CO_2_ fixation rate yields a primary production rate of ~0.6 µM d^-1^.


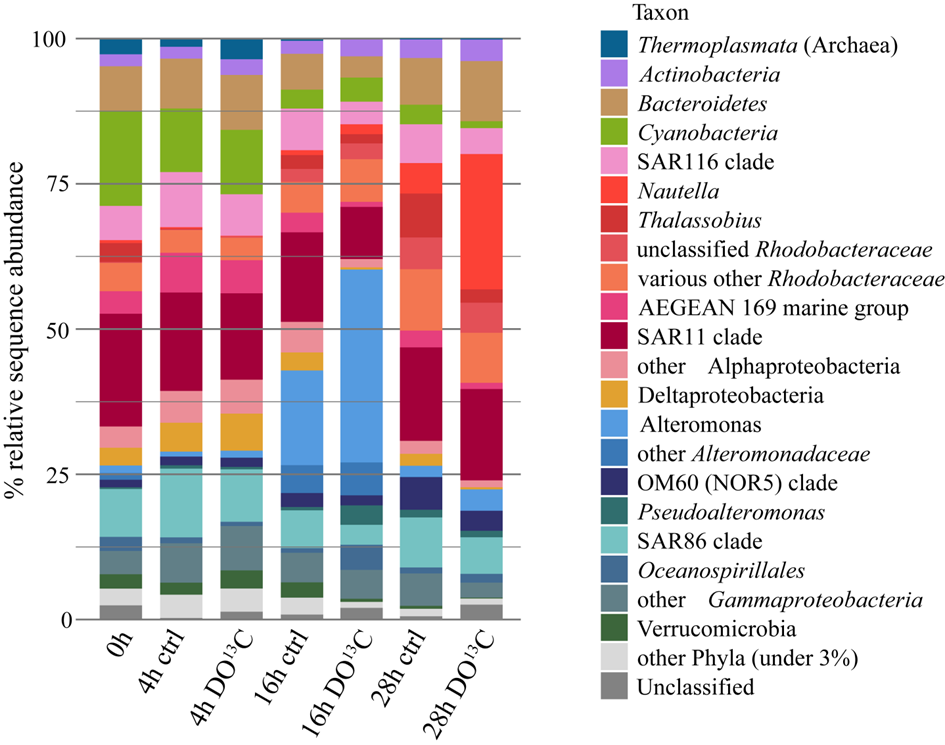


**Fig. S2.** The community composition from the DO^13^C amended and DI^13^C amended control incubations over time.


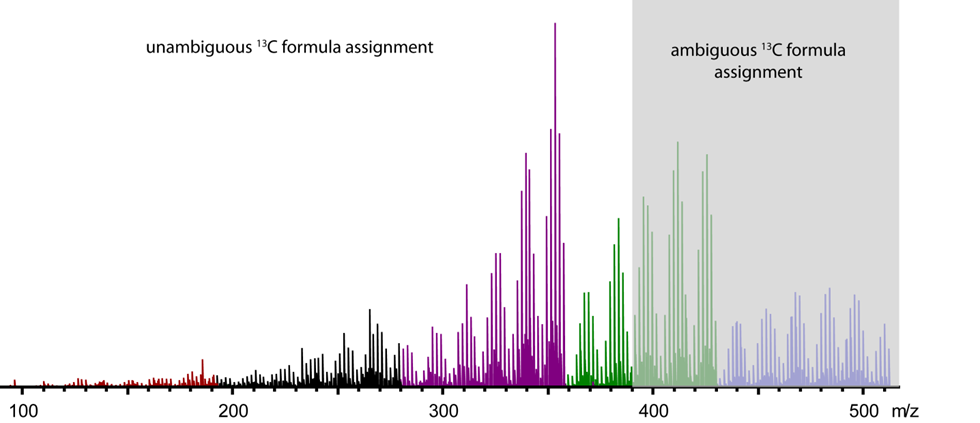


**Fig. S3.** The 5 different subdivisions of the mass window from FT-ICR-MS. The mass window was separated into subdivisions spectra, in order to raise the detection limits for single masses. All windows were used to identify DO^13^C molecules. However, unambiguous molecular formulas could only be assigned up to mass of ~391 m/z . The grey area denotes the mass range for which we could not calculate a unique ^13^C labelled chemical composition based on the mass of the each peak.


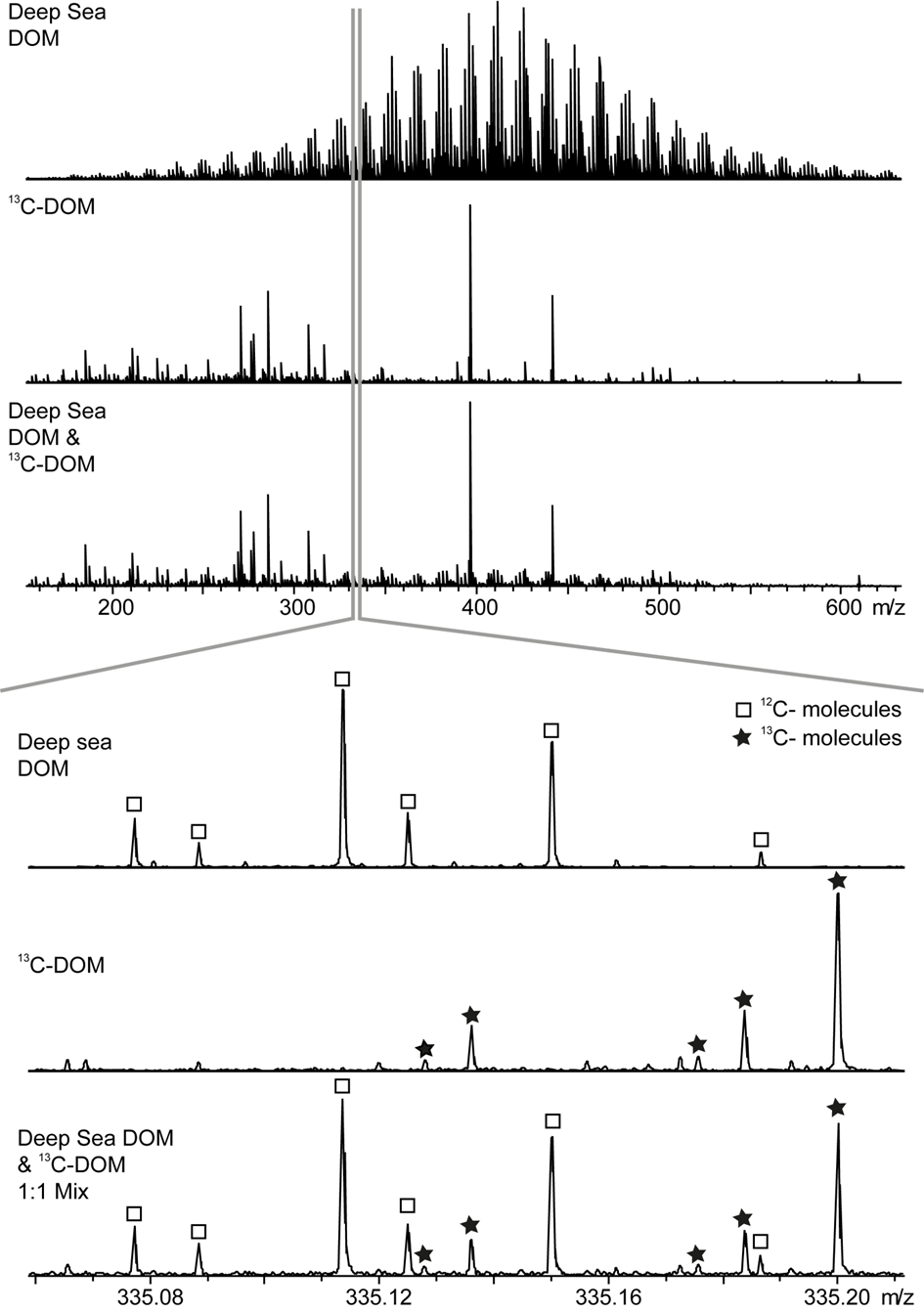


**Fig. S4.** Overview of mass spectra from an SPE extracted seawater standard from the North Equatorial Pacific Intermediate Waters (NEqPIW) sampled at station (Nelha), SPE extracted ^13^C-DOM, and a 1:1 mixture of the two. This demonstrates that the SPE-DO^13^C extract is clearly detectable and the ^12^C and ^13^C peaks could be distinguished from each other in the mixture of SPE-DO^13^C and deep sea SPE-DOM. The top three graphs show the complete mass spectra. The bottom three graphs show an excerpt from mass 335. The white squares mark the peaks that are present in the seawater while the black stars come from the SPE-DO^13^C and contain no ^12^C atoms.


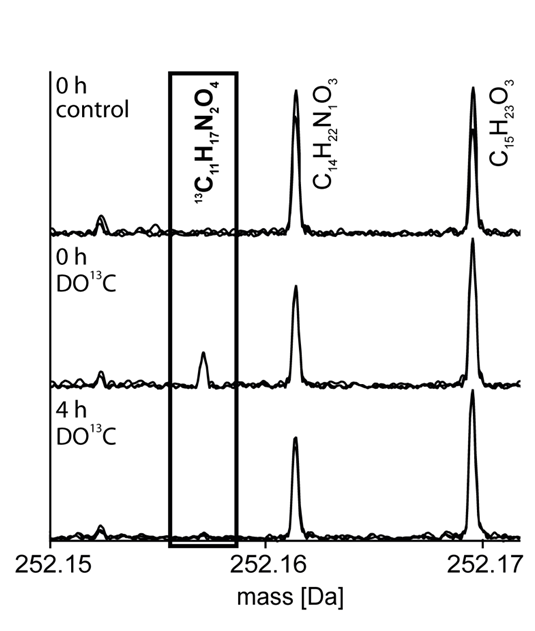


**Fig. S5**. Excerpts from FT-ICR-MS mass spectra showing the 252 Da mass range from triplicate measurements of three samples taken at different time points. The top mass spectra belongs to the unamended control, the middle spectra is the DO^13^C amended sample taken at 0h and the bottom is the DO^13^C amended sample taken at 4h. The highlighted region shows the appearance of a 100% ^13^C labelled molecule after ^13^C-DOM addition that was no longer present at 4h.


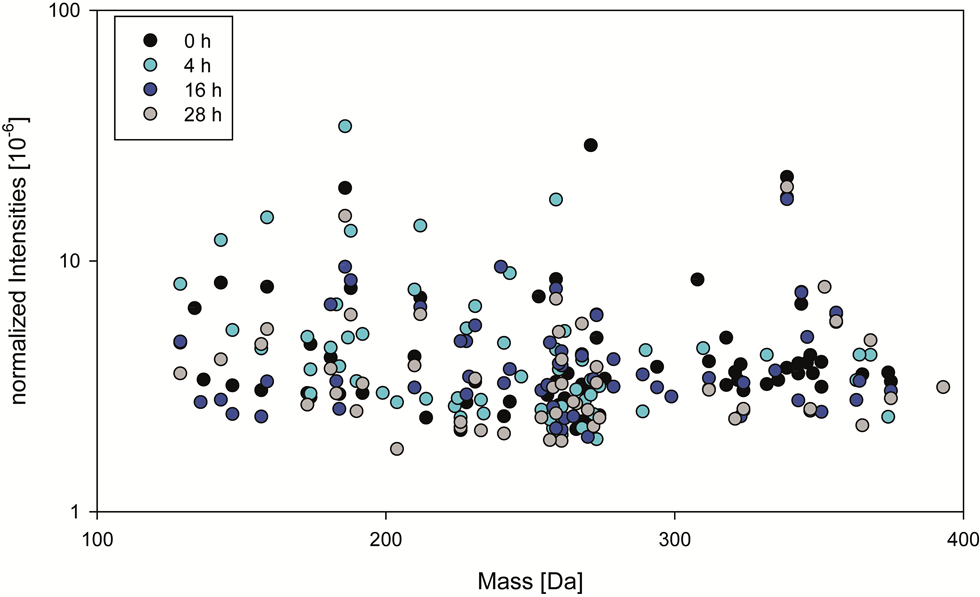


**Fig. S6.** The distribution of the masses of all DO^13^C identified compounds for 0h (black), 4h (cyan), 16h (blue) and 28h (grey). The amended ^13^C-DOM contained such molecules and possibly colloidal material. Previous reports have shown that smaller size classes of DOM include decomposition products from larger organic molecules (*1*). Hence, if the molecules larger than 391 Da were decomposed, degradation products of these molecules would have entered our window of detection as “new molecules”.


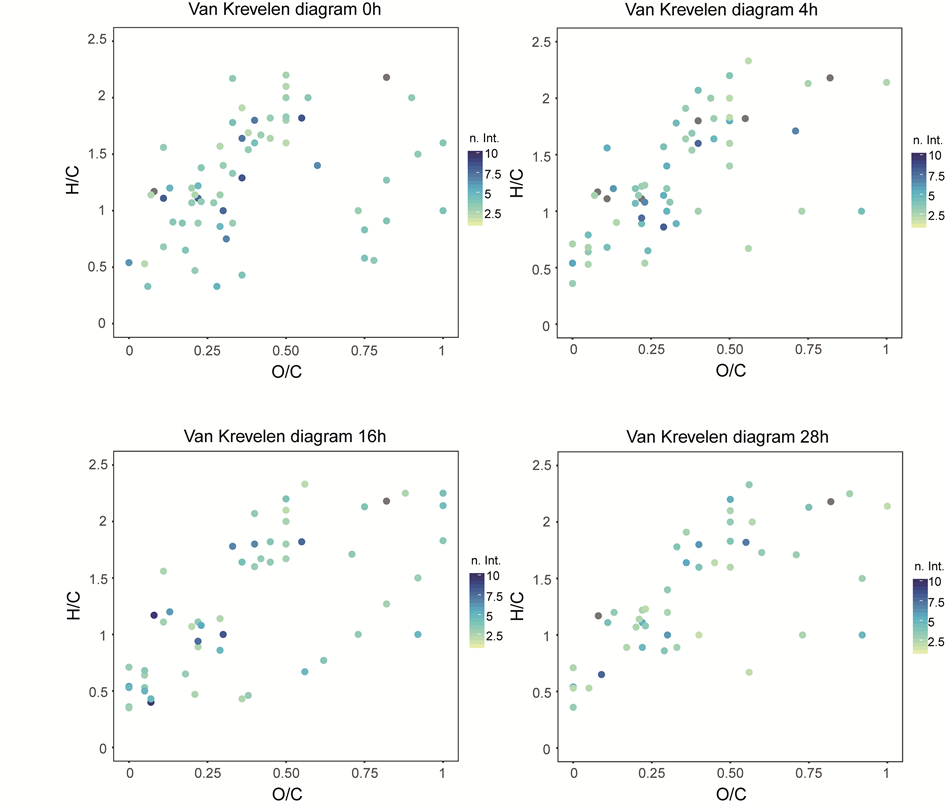


**Fig. S7.** Van Krevelen diagrams from each time point showing all molecules and their respective intensity (10^-6^). Note that molecules with a normalized intensity bigger than 10^-6^ are grey.

Table S1. Molecular formula of all ^13^C labelled molecules detected during the incubation. Numbering is the same as in Fig. 2.

| No. | Molecular Formula | No. | Molecular Formula | No. | Molecular Formula |
| --- | --- | --- | --- | --- | --- |
| 1 | ^12^C_4_^13^C_7_H_24_O_9_S_1_ | 37 | ^12^C_10_^13^C_12_H_8_O_0_N_2_ | 73 | ^12^C_2_^13^C_16_H_6_O_5_ |
| 2 | ^13^C_12_H_14_O_1_ | 38 | ^12^C_2_^13^C_6_H_17_O_6_N_1_S_1_ | 74 | ^13^C_18_H_6_O_1_N_4_ |
| 3 | ^13^C_11_H_20_O_6_ | 39 | ^12^C_7_^13^C_10_H_12_N_2_ | 75 | ^12^C_4_^13^C_8_H_10_O_9_N_1_P_1_ |
| 4 | ^13^C_9_H_10_O_1_ | 40 | ^12^C_3_^13^C_4_H_15_O_7_N_1_S_1_ | 76 | ^12^C_3_^13^C_9_H_7_O_9_N_3_ |
| 5 | ^13^C_9_H_10_O_2_ | 41 | ^12^C_2_^13^C_7_H_6_O_5_N_4_ | 77 | ^12^C_2_^13^C_8_H_10_O_10_N_1_P_1_ |
| 6 | ^13^C_10_H_10_O_3_ | 42 | ^12^C_4_^13^C_6_H_20_O_5_S_1_ | 78 | ^12^C_3_^13^C_8_H_10_O_9_N_1_P_1_ |
| 7 | ^13^C_10_H_18_O_4_ | 43 | ^12^C_3_^13^C_6_H_21_O_5_N_1_S_1_ | 79 | ^12^C_3_^13^C_7_H_16_O_10_N_1_P_1_ |
| 8 | ^12^C_11_^13^C_13_H_13_N_3_ | 44 | ^13^C_11_H_20_O_5_N_2_ | 80 | ^13^C_13_H_18_O_3_N_2_ |
| 9 | ^13^C_15_H_18_O_2_N_2_ | 45 | ^13^C_9_H_11_O_2_N_1_ | 81 | ^13^C_9_H_5_O_7_N_3_ |
| 10 | ^13^C_7_H_6_O_2_ | 46 | ^12^C_8_^13^C_13_H_19_O_3_N_3_ | 82 | ^13^C_6_H_13_O_2_N_1_ |
| 11 | ^13^C_10_H_16_O_4_ | 47 | ^12^C_2_^13^C_17_H_13_O_2_N_3_ | 83 | ^12^C_3_^13^C_7_H_20_O_9_N_1_P_1_ |
| 12 | ^13^C_9_H_16_O_3_ | 48 | ^12^C_1_^13^C_12_H_20_O_5_ | 84 | ^12^C_1_^13^C_14_H_20_O_5_N_2_ |
| 13 | ^13^C_13_H_14_O_3_ | 49 | ^13^C_10_H_12_O_2_ | 85 | ^12^C_2_^13^C_13_H_16_O_4_N_1_P_1_ |
| 14 | ^13^C_15_H_16_O_3_ | 50 | ^13^C_14_H_16_O_1_ | 86 | ^12^C_1_^13^C_10_H_20_O_5_N_2_ |
| 15 | ^12^C_2_^13^C_9_H_18_O_4_N_1_P_1_ | 51 | ^13^C_14_H_22_O_4_ | 87 | ^12^C_1_^13^C_9_H_10_O_3_ |
| 16 | ^13^C_9_H_8_O_2_ | 52 | ^13^C_13_H_22_O_5_ | 88 | ^13^C_17_H_11_O_4_N_1_ |
| 17 | ^12^C_4_^13^C_7_H_11_O_8_N_1_S_1_ | 53 | ^12^C_1_^13^C_13_H_6_O_5_N_4_ | 89 | ^12^C_2_^13^C_17_H_15_O_1_N_1_ |
| 18 | ^12^C_4_^13^C_6_H_22_O_5_S_1_ | 54 | ^13^C_17_H_11_O_3_N_1_ | 90 | ^13^C_13_H_14_O_4_ |
| 19 | ^13^C_11_H_18_O_5_ | 55 | ^12^C_3_^13^C_8_H_14_O_9_N_1_P_1_ | 91 | ^13^C_9_H_18_O_4_ |
| 20 | ^12^C_1_^13^C_18_H_10_O_1_ | 56 | ^13^C_19_H_9_O_4_P_1_ | 92 | ^13^C_11_H_18_O_4_ |
| 21 | ^13^C_9_H_14_O_1_ | 57 | ^13^C_12_H_20_O_5_ | 93 | ^13^C_13_H_20_O_5_ |
| 22 | ^13^C_8_H_16_O_4_ | 58 | ^12^C_5_^13^C_13_H_16_O_3_N_2_ | 94 | ^13^C_10_H_14_O_5_ |
| 23 | ^13^C_14_H_16_O_4_ | 59 | ^12^C_1_^13^C_13_H_28_O_8_N_2_ | 95 | ^12^C_4_^13^C_9_H_7_O_3_N_1_ |
| 24 | ^13^C_11_H_20_O_5_ | 60 | ^12^C_2_^13^C_16_H_17_O_4_P_1_ | 96 | ^12^C_5_^13^C_10_H_6_O_1_N_2_ |
| 25 | ^13^C_10_H_18_O_5_ | 61 | ^12^C_4_^13^C_11_H_31_O_6_N_1_S_1_ | 97 | ^12^C_9_^13^C_13_H_11_O_1_N_3_ |
| 26 | ^12^C_1_^13^C_17_H_22_O_4_N_2_ | 62 | ^12^C_9_^13^C_13_H_14_O_1_N_4_ | 98 | ^12^C_4_^13^C_10_H_6_O_1_N_2_ |
| 27 | ^12^C_1_^13^C_11_H_22_O_6_ | 63 | ^12^C_1_^13^C_18_H_13_O_1_N_1_ | 99 | ^12^C_3_^13^C_5_H_18_O_8_S_1_ |
| 28 | ^13^C_10_H_14_O_3_ | 64 | ^13^C_10_H_12_O_3_ | 100 | ^13^C_13_H_10_O_8_N_2_ |
| 29 | ^13^C_9_H_8_O_3_ | 65 | ^13^C_13_H_16_O_3_ | 101 | ^12^C_2_^13^C_4_H_11_O_6_N_1_S_1_ |
| 30 | ^13^C_14_H_16_O_3_N_2_ | 66 | ^13^C_10_H_10_O_4_ | 102 | ^12^C_4_^13^C_9_H_6_O_5_N_2_ |
| 31 | ^12^C_4_^13^C_7_H_21_O_4_N_3_ | 67 | ^12^C_5_^13^C_10_H_8_N_2_ | 103 | ^12^C_8_^13^C_15_H_8_ |
| 32 | ^13^C_10_H_16_O_5_ | 68 | ^12^C_1_^13^C_7_H_18_O_7_S_1_ | 104 | ^13^C_6_H_10_O_3_ |
| 33 | ^12^C_3_^13^C_9_H_18_O_11_N_2_ | 69 | ^13^C_14_H_18_O_5_N_2_ | 105 | ^12^C_8_^13^C_15_H_15_O_2_N_1_ |
| 34 | ^12^C_3_^13^C_7_H_21_O_5_N_3_ | 70 | ^13^C_11_H_18_O_4_N_2_ | 106 | ^13^C_15_H_26_O_9_N_2_ |
| 35 | ^13^C_7_H_12_O_5_ | 71 | ^12^C_1_^13^C_15_H_12_O_5_N_1_P_1_ |  |  |
| 36 | ^12^C_4_^13^C_8_H_12_O_11_N_2_ | 72 | ^13^C_5_H_7_O_3_N_1_ |  |  |

**Supplementary references**

1. R. Benner, R. M. W. Amon, The Size-Reactivity Continuum of Major Bioelements in the Ocean. *Annu. Rev. Mar. Sci.* **7**, 185-205 (2015).
